# Super-resolution for localizing electrode grids as small, deformable objects during epilepsy surgery using augmented reality headsets

**DOI:** 10.1007/s11548-025-03401-5

**Published:** 2025-06-19

**Authors:** Hizirwan S. Salim, Abdullah Thabit, Sem Hoogteijling, Maryse A. van ’t Klooster, Theo van Walsum, Maeike Zijlmans, Mohamed Benmahdjoub

**Affiliations:** 1 High Performance Compute & Visualization, SURF bv, Utrecht, The Netherlands; 2 Department of Radiology & Nuclear Medicine, Biomedical Imaging Group Rotterdam, Erasmus MC, Rotterdam, The Netherlands; 3 Department of Oral & Maxillofacial surgery, Erasmus MC, Rotterdam, The Netherlands; 4 Department of Neurology and Neurosurgery, UMC Utrecht Brain Center Utrecht, Part of ERC EpiCare, University Medical Center Utrecht, Utrecht, The Netherlands; 5 Stichting Epilepsie Instellingen Nederland (SEIN), Hoofddorp, The Netherlands

**Keywords:** Intra-operative electrocorticography, Augmented reality, Computer-assisted surgery, Artificial intelligence, Epilepsy surgery, Pose estimation, Instrument tracking

## Abstract

**Purpose:**

Epilepsy surgery is a potential curative treatment for people with focal epilepsy. Intraoperative electrocorticogram (ioECoG) recordings from the brain guide neurosurgeons during resection. Accurate localization of epileptic activity and thus the ioECoG grids is critical for successful outcomes. We aim to develop and evaluate the feasibility of a novel method for localizing small, deformable objects using augmented reality (AR) head-mounted displays (HMDs) and artificial intelligence (AI). AR HMDs combine cameras and patient overlay visualization in a compact design.

**Methods:**

We developed an image processing method for the HoloLens 2 to localize a 64-electrode ioECoG grid even when individual electrodes are indistinguishable due to low resolution. The method combines object detection, super-resolution, and pose estimation AI models with stereo triangulation. A synthetic dataset of 90,000 images trained the super-resolution and pose estimation models. The system was tested in a controlled environment against an optical tracker as ground truth. Accuracy was evaluated at distances between 40 and 90 cm.

**Results:**

The system achieved sub-5 mm accuracy in localizing the ioECoG grid at distances shorter than 60 cm. At 40 cm, the accuracy remained below 2 mm, with an average standard deviation of less than 0.5 mm. At 60 cm the method processed on average 24 stereo frames per second.

**Conclusion:**

This study demonstrates the feasibility of localizing small, deformable objects like ioECoG grids using AR HMDs. While results indicate clinically acceptable accuracy, further research is needed to validate the method in clinical environments and assess its impact on surgical precision and outcomes.

## Introduction

**Fig. 1:**
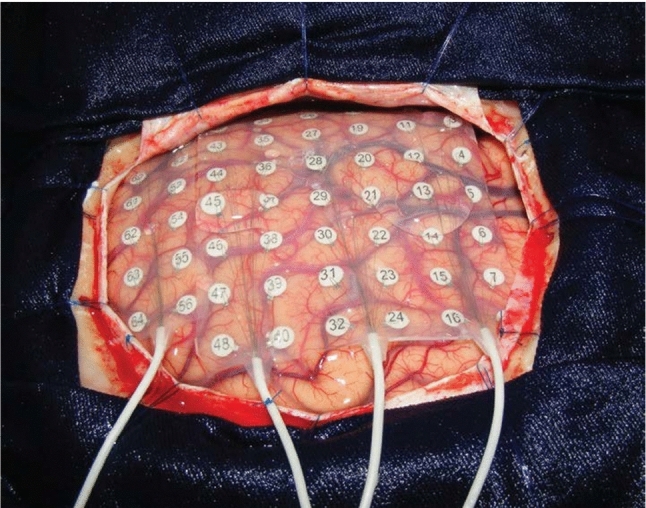
An example of an ioECoG placed on a patient’s brain, adapted from [12]

Epilepsy is a neurological disorder affecting approximately 50 million people worldwide, making it one of the most prevalent chronic neurological conditions. In around 30% of people with epilepsy, anti-seizure medication fails to control seizures. Epilepsy surgery offers a potential lifelong curative treatment for people with focal—epilepsy originating from one spot in the brain—refractory epilepsy. Surgical success depends on precise delineation and complete resection of epileptic tissue. During so-called tailored epilepsy surgery, an EEG electrode grid is placed directly on the cortex (i.e., intraoperative electrocorticography [ioECoG]) to localize epileptic tissue based on pathological ioECoG activity (see Fig. 1) [6]. To pinpoint the source of the pathological activity in the brain, accurate localization of the ioECoG grid is crucial. This is challenging as the ioECoG grid is deformable, small, and may be partially obscured, as it can be slid under the skull [14].

In current clinical practice, localization of ioECoG position is merely done by means of visual inspection [10]. Previously described research on ioECoG grid localization approaches involves matching digital photographs of the ioECoG grids to a presurgical 3D MRI. Pieters et al. compared seven methods for accuracy against the photograph-based technique, which was considered the most accurate. The top-performing approach, a semi-automatic grid-partitioning method, achieved a mean error of 2.0 mm and a maximum error of 6.4 mm, with the mean falling below our defined clinically acceptable threshold of 5 mm. However, their study also revealed significant limitations in both the photograph-based and semi-automatic methods, such as prolonged registration times-sometimes extending to several hours-and the need for specialized expertise. Moreover, these methods are restricted to electrode grid points that are fully visible and not occluded by the skull. Consequently, developing a method that remains within clinically acceptable thresholds for accuracy, operates more quickly, and requires less expertise could greatly improve epilepsy surgery outcomes.

Recently, augmented reality (AR) has emerged as a promising tool for enhancing surgical navigation by overlaying real-time 3D information onto the surgeon’s field of view [3]. AR head-mounted displays (HMDs), equipped with integrated cameras, offer the ability to localize rigid medical instruments and devices when equipped with markers [7]. Unlike handheld devices or external monitors, AR HMDs offer a compact form factor that enables hands-free interaction. This seamless integration of information into the surgeon’s workflow can improve efficiency, ergonomics, and accuracy during complex procedures [1, 3]. More importantly, common tracking devices in the operating room, such as optical and electromagnetic systems, are unsuitable for ioECoG grid localization because they rely on reflective materials, embedded coils, or time-consuming registration. Depth-measuring cameras are rarely available, and no widely accessible OR devices currently support automatic ioECoG grid detection. Although AR HMDs like the HoloLens 2 are not yet standard, there are FDA-approved solutions for neurosurgery and AR HMDs offer dual functionality for both visualization and localization of instruments [5]. Stereo-camera microscopes with digital overlays provide similar capabilities but have several limitations including high cost, workflow disruption due to bulkiness and sterile packing, and potential interference with ECoG recordings. However, detecting small, deformable objects like ioECoG grids with AR HMDs requires specialized approaches that current AR systems have yet to fully address.

Current vision-based localization methods are limited to rigid markers or markers of sufficient size when using low-resolution sensors to track them with clinically acceptable accuracy [7]. AI techniques, particularly deep learning, show enormous potential in overcoming the limitations of traditional optical tracking methods. Recent advancements in AI outside of surgical applications include the use of neural networks for object detection, super-resolution to enhance image quality, and 2D pose estimation to estimate poses in images. AI has been explored only to a limited extent for intraoperative guidance in brain surgery [8], and not at all when combined with AR HMDs.

We propose a novel method that combines an AR HMD with AI to address challenges posed by low-resolution sensors and deformable objects. By integrating AI-based object detection, super-resolution, and 2D pose estimation with traditional stereo vision, we aim to improve the accuracy and workflow of electrode grid detection and localization compared to manual photograph-based alignment, while enabling real-time AR visualization. The proposed method leverages synthetic data to address the common challenge of data scarcity in the medical domain. To the best of our knowledge, automatic localization of ioECoG grids, particularly using deep learning, has not been explored in previous research.

The primary objective is to develop and evaluate the feasibility of the proposed method. We assess its accuracy through a controlled experiment, comparing performance to a commercial NDI tracking system, which serves as the ground truth. Our findings provide valuable insights into the potential of AI and AR to enhance surgical precision and improve patient outcomes in epilepsy surgery.

## Methodology

**Fig. 2:**
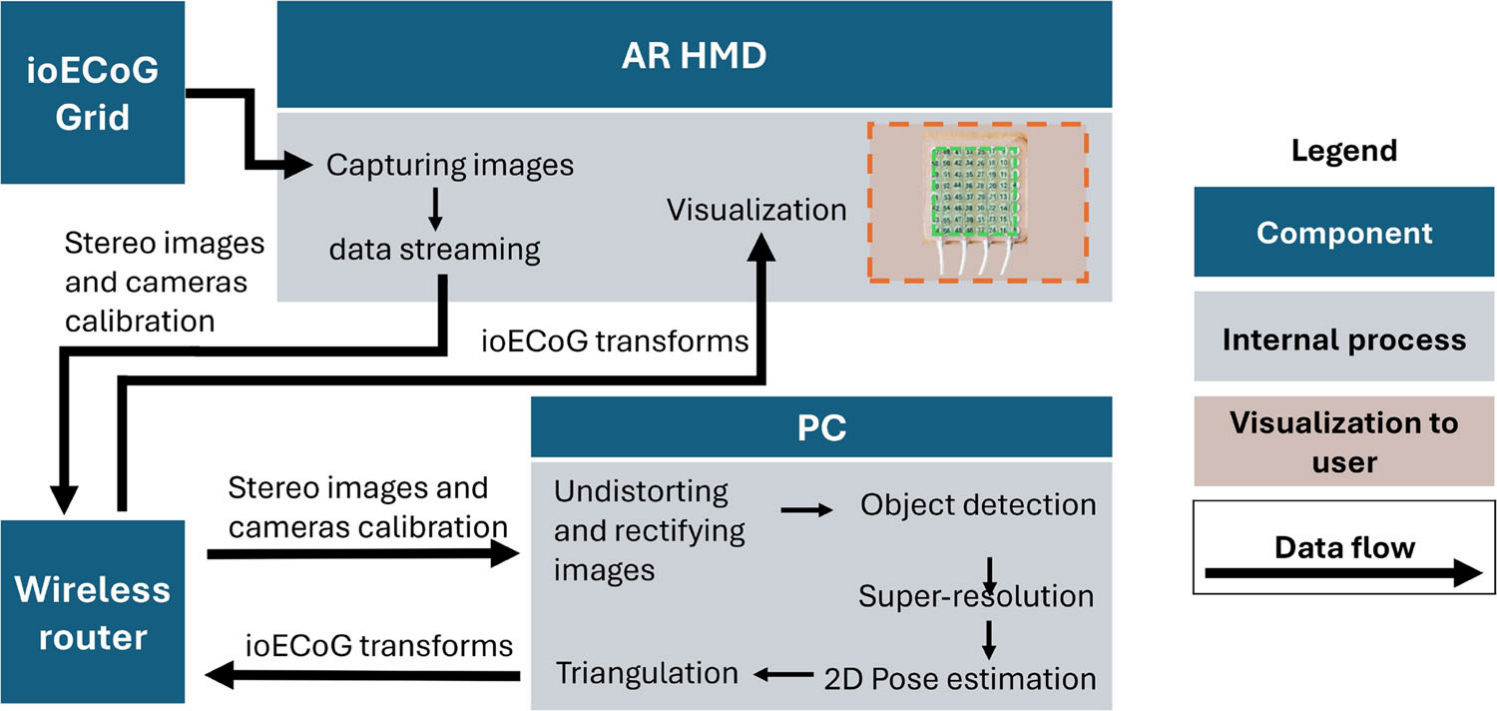
A schematic overview of the proposed system for ioECoG grid position estimation containing the interaction between the ioECoG grid, AR HMD, wireless router and the PC

The objective of our method is to accurately detect the center of each individual electrode disk of the ioECoG grid (64 electrodes, 5 mm inter-electrode distance). Figure 2 illustrates the proposed system, which includes an AR HMD, a PC, and the ioECoG itself. First step of this method is the wireless transmission of stereo image frames from the two front grayscale cameras of the HoloLens to the PC.

As part of the PC’s internal process, the first step is to apply image undistortion and rectification. This is necessary to correct the distortion inherent in camera optics, which would otherwise reduce the accuracy of the subsequent triangulation. Second, the ioECoG grid is identified and a bounding box is estimated, reducing the image resolution for the subsequent steps and eliminating irrelevant image context. The cropped images typically range from 22 to 60 pixels in width. Third, due to the low resolution of these images, which limits the ability to distinguish the contours of the disks and their centers, a super-resolution model is applied to upscale the image resolution by a factor of four. This enhancement allows the 2D pose estimation model to accurately locate the disks and their corresponding number in the physical grid as a fourth step. As a fifth and final step, the two 2D poses, derived of each stereo image, are used to perform triangulation, yielding the 3D positions of the ioECoG grid points. These steps are explained in more detail in the next sections.

**Fig. 3:**
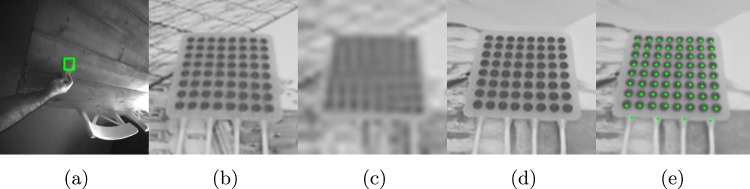
Data creation part for training the models, respectively: Real grayscale image with object and bounding box label (**a**), Blender render with size 80x80 pixels (**b**), 0.33x downscaled image to 26x26 pixels (**c**), upscaled image by 4x with trained super-resolution model (**d**), image and label that is used for training 2D pose estimation (**e**)

### Object detection

After undistorting and rectifying the images, the next step in localizing the ioECoG grid is to detect the object and estimate its bounding box. We trained a YOLOv8 model [9] using 400 images captured by the HoloLens 2 gray-scale stereo cameras, which have 640x480 pixels. An example can be found in Fig. 3a. The bounding boxes of the ioECoG grids were manually annotated by the first author (HSS). 80% of the images were used for training, and 20% were used for validation. Once the object was detected and the bounding box was estimated, it was used to crop the image, reducing the area for further processing in the subsequent steps.

### Image upscaling via super-resolution

Enhancing the image quality using a super-resolution technique is necessary since the cropped images of the ioECoG grid can be as small as 20x20 pixels. This size is insufficient for accurate 2D pose estimation. The super-resolution model was trained using 90,000 synthetically generated images. These synthetic images were created in Blender (www.blender.org), simulating randomized angles, backgrounds, lighting conditions, and deformations of the ioECoG grid. Additionally, in this step, we automatically generated the ground-truth 2D pose labels in Blender. We did this by projecting the center of each 3D electrode disk onto the image plane. These labels were then used in the next step for training the 2D pose estimation model.

**Fig. 4:**
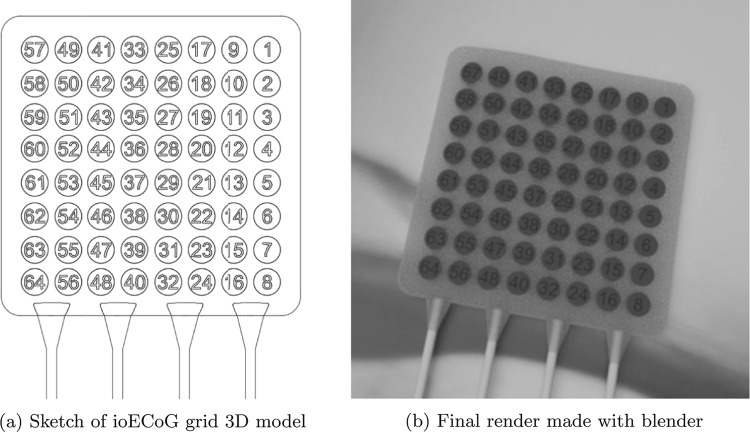
A side-by-side comparison of a sketch of the ioECoG grid model and the final render with maximum resolution made in blender

To train the super-resolution model, we created downsampled versions of the synthetic images with 75%, 50%, and 33% of the original resolution, generating a total of 360,000 images. The resolutions of the training dataset ranged between 26x26 pixels and 260x260 pixels. We used a pre-trained REAL-ESRGAN [13] model and fine-tuned its weights with our dataset. Figure 4 shows an illustration of the digital 3D model of the ioECoG grid, along with a render at the maximum resolution of 260x260 pixels.

### 2D pose estimation

After upscaling the grayscale images four times using the super-resolution model, 2D pose estimation is performed. For the training data of the 2D pose estimation model, we used the 0.33x downsampled renderings and utilized the fine-tuned super-resolution model to upscale these images, as this step is also present in our proposed method. Since the training data consisted of synthetically generated renders, we could extract the 2D pose labels directly from Blender, as described earlier.

We fine-tuned a pre-trained YOLOv8-Pose model [9] using our dataset. The model outputs the position of each individual ioECoG electrode and the four cables, providing 68 pixel coordinates for each stereo image. The cables, also referred to as tails, help the model orient itself correctly because the ioECoG grid points are arranged in a symmetrical square pattern. This symmetry allows the grid to be rotated 90 degrees, and without proper orientation, the wrong electrode numbers may be assigned to individual electrodes. By triangulating the corresponding points of each stereo image, the 3D positions of the ioECoG grid were computed. An example of one of the training images with labels is shown in Fig. 3.

## Experiment & results

**Fig. 5:**
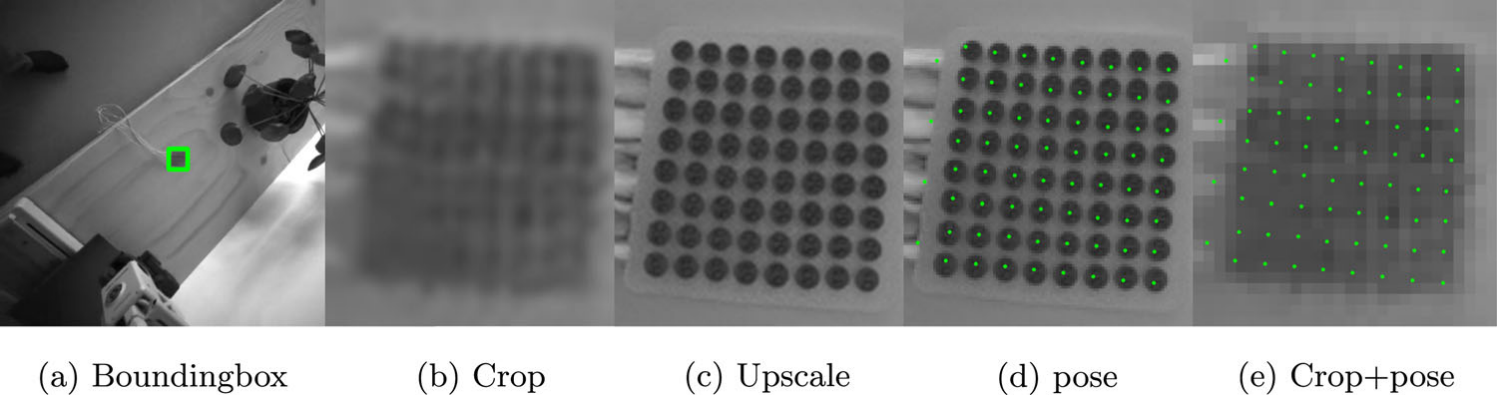
The image processing pipeline for one of the stereo images

For qualitative testing on real images, the proposed ioECoG grid localization method was evaluated on test images acquired from the HoloLens 2’s stereo cameras. Figure 5 shows sample test images after ioECoG object detection, cropping, super-resolution upscaling and 2D pose estimation. Each step of the pipeline was independently validated after training by ensuring detection of the ioECoG grid’s bounding box using sample images, assessing visually the quality of super-resolution images, and verifying that the estimated grid points positions were within the disks. Once the outputs appeared visually satisfactory, we advanced to experimental testing of the complete pipeline for localizing the ioECoG grid. The qualitative test demonstrated the ability of the proposed approach to locate the ioECoG grid electrodes on real images. To further assess the tracking accuracy quantitatively, an experiment was conducted to compare the estimated pose of the ioECoG Grid using the HoloLens 2 against an optical tracking system. The setup and results of the conducted evaluation experiment are further detailed in the following subsections.

### Experimental setup

The evaluation experiment aimed to assess the tracking accuracy of locating the ioECoG grid under a single pose, where the ioECoG grid is static, flat and facing the HMDs camera. Given that the ground truth location of the ioECoG grid relative to the AR HMD is not available, relative-poses assessment was conducted. An NDI Polaris Vega optical tracking system was manually calibrated to track the ioECoG grid. This calibration was done using a trackable pointer and pinpointing the individual ioECoG disks. The relative pose measurements reported by the NDI served as ground truth and compared to that reported by the HoloLens 2.

The experimental setup consisted of the following components: an ioECoG grid (Ad-Tech 64-electrode ioECoG grid with a 5 mm interelectrode distance and 4 mm electrode disks), a Microsoft HoloLens 2 as AR HMD (main application in Unity (www.unity.com), with HL2SS [4] being used to stream the camera images to a PC (CPU: Intel i9 12900K, GPU: NVidia RTX 3090, RAM: 32GB), and an NDI Polaris Vega with retroreflective markers for ground truth measurements.

**Fig. 6:**
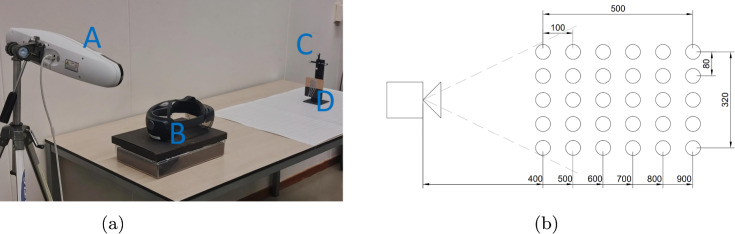
The experimental setup for the evaluation is illustrated in part (**a**) of the figure, which includes an NDI Vega Polaris (**A**), a HoloLens 2 (**B**), a retroreflective marker (**C**) and an ioECoG grid (**D**). Part (**b**) shows a schematic representation of the measurement grid for 30 predefined positions relative to the HoloLens 2, with units in millimeters

For the relative pose assessment, a measurement grid was defined such that the distance between the HoloLens 2 and the ioECoG grid varied between 40 and 90 cm, which covers the working distance expected during surgery (arm’s length). The width of the measurement grid was designed so that, at the closest distance (40 cm), the ioECoG grid would just fit within the field of view of the stereo cameras. This configuration resulted in a working area of 32x50 cm, which is deemed sufficient for the intended clinical use (see Fig. 6).

To track the ioECoG grid by the NDI optical tracking system, the ioECoG grid was fixated to a retroreflective NDI marker. The center of the ioECoG grid points was manually annotated with the NDI pointer to locate their positions in the attached NDI marker’s coordinate system.

The experiment was designed to assess the accuracy of the method in tracking the movement of the individual ioECoG grid electrodes across the positions of the measurements grid. The ioECoG grid was manually moved to the 30 predefined positions. At each position, the grid was rotated manually so that it faced the HoloLens 2 directly and 150 data points were recorded for measurements. The whole experiment was repeated five times over the 30 predefined positions.

The closest central position (40 cm) was chosen as a reference position. It was assumed to yield the highest accuracy for the ioECoG grid detection using the HoloLens 2. The relative change in position (Euclidean distance) between the ioECoG grid when placed on the reference position and the other positions (*n*=29) was computed. This relative change was compared between the NDI and the HoloLens 2 for every ioECoG grid point. The difference in relative change between the NDI and the HoloLens 2 represents our measured error, which we compare to our clinically acceptable threshold of 5 mm. A visual representation of the experimental setup is shown in Fig. 6. To ensure correct detection and localization of the ioECoG grid, a few steps were taken to remove outliers (false positives). First, only measurements where a single bounding box was detected in each stereo frame were considered. Second, since the ioECoG grid is assumed to remain static, any measurements deviating significantly from the median position were treated as outliers. Specifically, a threshold of 10 cm from the median was applied to exclude these false positives. The false positive rate is therefore defined as the proportion of measurements falling outside the specified threshold, considering only frames in which a bounding box was detected in both stereo frames. To evaluate the error distribution across the ioECoG grid points, we calculate the total error for each electrode across all measurement positions and then normalize the results to assess their uniformity. Normalization is achieved by dividing each electrode’s errors by the maximum error observed for a single electrode.

The experiment took place in an environment with minimal external interference. To ensure consistency, the laboratory was free of natural light, and all measurements were taken under stable artificial lighting conditions.

### Performance evaluation

**Fig. 7:**
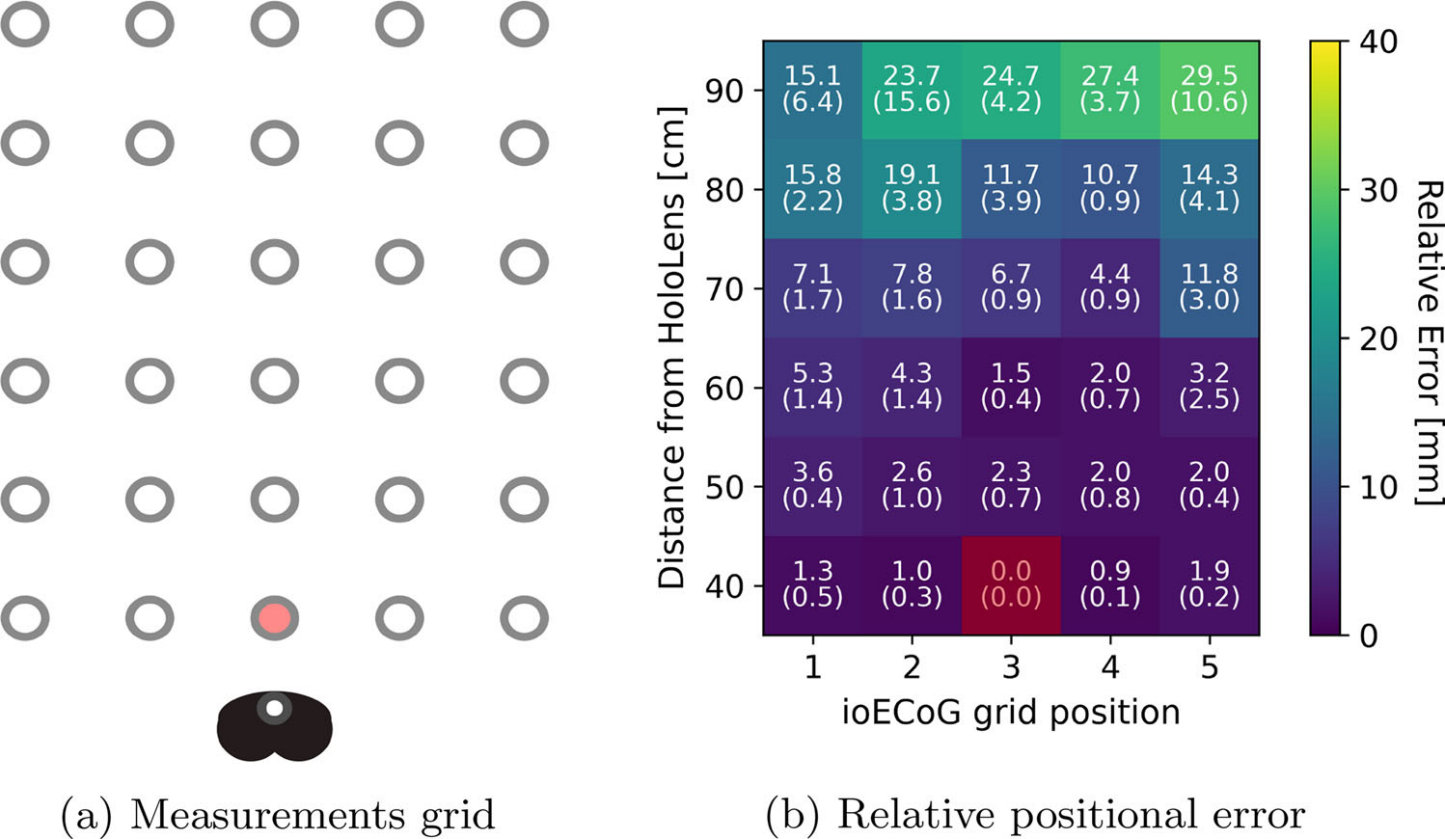
Relative positional error over the measurements grid for all 30 predefined positions with respect to the reference position (red). The values in the heat map represent the mean and standard deviation of the averaged relative-pose error of the ioECoG grid at each position

**Fig. 8:**
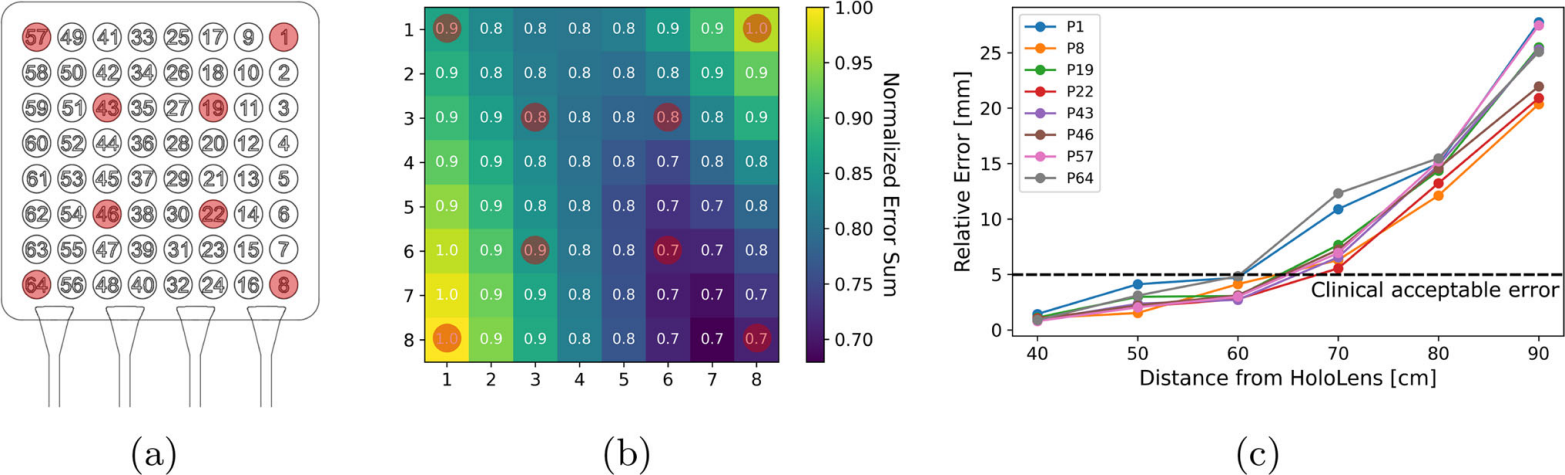
Positional error for the individual ioECoG grid points. **a** The ioECoG grid. **b** Heatmap for the normalized error sum of the 64 ioECoG grid points over all 30 positions. **c** Averaged positional error for eighth individual ioECoG grid electrodes (indicated in red in **a** and **b**) for 40-90 cm distances from the HoloLens 2

Following the experimental setup discussed in "Experimental setup" Section, the average relative error for all 64 grid points across all five runs for each position is shown in Fig. 7. The average relative error increases as the distance from the HoloLens 2 increases but remains less than 5 mm on average up to 60 cm. After 60 cm distance, the relative error increases to centimeter levels and the reported position of the ioECoG grid becomes noisy, with a higher standard deviation across the 150 recorded frames. Figure 8 shows the distribution of the relative error for each of the 64 grid points across all 30 predefined positions (Fig. 8b) and the relative error of some selected ioECoG grid points over multiple distances from the HoloLens 2. The relative error indicates a non-homogeneous distribution across the 64 electrode grid points (Fig. 8), where points close to the bottom left corner and up right corner reporting the highest error. Moreover, with chosen threshold of 10 cm from the median, we observed no false positives for measurements of 60 cm or less. However, for measurements of 70 cm and above, the average false positive rate was 6%. The end-to-end processing time depends on the distance to the ioECoG grid. At 60 cm, the average processing time is 41.9 ms, equating to approximately 24 stereo frames per second.

## Discussion

This study investigated the feasibility of integrating AR HMDs with AI for the precise localization of small, deformable objects, specifically ioECoG grids used during epilepsy surgery. Our approach, combining object detection, super-resolution, 2D pose estimation, and stereo triangulation with an AR HMD (HoloLens 2), has shown promising results in a controlled experimental setting. The super-resolution and 2D pose estimation models were successfully trained on purely synthetic data. The system achieved sub-5 mm accuracy below 60 cm distances. At 40 cm, this accuracy improved to less than 1.9 mm, with an average standard deviation of 0.5 mm. The estimation of individual electrodes did not demonstrate uniform performance, with the top right and bottom left electrodes of the ioECoG grid showing the poorest results.

### Performance analysis

Positional errors increase with distance, particularly with the low resolution (640x480) and wide-angle lenses of the HoloLens 2 cameras. As distance grows, image details diminish, forcing the super-resolution and 2D pose estimation model to rely more on approximations. While our method cannot match the sub-millimeter accuracy of the NDI Polaris Vega with manual registration, it remains within a clinically acceptable margin of 5 mm for localizing ioECoG grids up to 60 cm away.

As shown in Fig. 8b). This may stem from bias in the model’s pose estimation, though further investigation is needed.

Other localization methods, such as recursive grid partitioning, have demonstrated competitive results, with a reported mean localization error of 2.0 mm [10]. However, this approach is time-consuming and requires specialized expertise. In contrast, our AR-based method offers a more efficient, less operator-dependent solution.

GridLoc, an alternative ioECoG grid localization method based on signal analysis, achieved an overall accuracy of 1.94 mm [2]. However, GridLoc assumes a known and small inter-electrode distance, a large enough grid coverage, is time consuming and relies on pre-surgical angiographic data and radioactive MRI imaging.

Both methods lack real-time feedback and show the ioECoG electrodes in the MRI scan or on a 3D model, without a direct way to translate to the operating field. This limitation may reduce their utility for neurosurgeons, who could benefit from real-time overlays directly onto the patient. The latter is only possible by integrating AR technology. This enhanced visualization, which might also be utilized to visualize the epileptic area, may result in improved workflow, shorter interventions and increased ergonomics for this type of surgery.

### Limitations and future directions

Despite promising results, several limitations must be addressed in future work. First, our experiments were conducted in a controlled setting, lacking the complexities present in operating rooms. Factors such as brain deformation, grid obscuration by the skull, and tissue appearance variations could affect accuracy. Additionally, the controlled setup had static ceiling lights with no shadows on the grid, which may differ in the OR. However, we expect adequate lighting in the OR, and since our synthetic data account for various lighting conditions, we anticipate minimal impact from lighting variations.

Second, this study did not evaluate different ioECoG grid deformations, as the grid was kept static to minimize variables. However, the AI models are not restricted to specific deformations, as long as the data are similar to the training set, offering an advantage over methods like grid-fitting. Incorporating more varied deformations in future synthetic data could improve the method’s ability to handle greater variations. Future work should explore these deformations to assess the robustness of the models.

Third, we measured relative accuracy. While this aligns with metrics used in comparable studies [7], additional testing is needed to determine absolute accuracy in a clinical setting. Given the submillimeter accuracy of NDI, we expect any error given compared to the ground truth to be negligible.

Fourth, in our evaluation, everything is considered static. While the ioECoG grid can be assumed to be static in a clinical context due to the head being fixed, some minimal movement of the HMD may occur. Since this method focuses on registration rather than tracking, we expect the impact of this movement to be minimal. Any significant HMD movement can be addressed by filtering out images where the acceleration exceeds a certain threshold.

The method could be improved by adding more sanity checks to minimize false positives. For instance, the depth camera could provide distance estimates in centimeters, serving as a threshold for filtering. Additionally, with the use of an AR HMD, the detected positions can be visualized, enabling the neurosurgeon to quickly and intuitively assess the results.

Finally, the results can be further refined by integrating additional sensors, such as an RGB camera, refining pose detection to the circle centers, or incorporating prior knowledge. For example, future studies could explore integrating pre-surgical imaging (e.g., MRI or cone beam CT) to improve localization accuracy. Refinement of the result can be achieved by combining this method with the MRI-based method of Trotta et al., which can be automated by utilizing the proposed method in this work to get an initial prediction [11].

### Clinical implications

Accurate real-time localization of ioECoG grids using AR HMDs has significant clinical implications for epilepsy surgery. It could enable more precise mapping of epileptic tissue, improving resection success rates and reducing seizure recurrence. The system’s non-disruptive nature and the ability to directly visualize ioECoG data via the HoloLens 2 could enhance surgical workflows and potentially shorten procedure time. As AR HMDs become more integrated into neurosurgery, leveraging this existing platform allows for smooth implementation. Additionally, this system could be adapted for other surgical applications requiring precise localization of small, deformable objects using synthetic data.

## Conclusion

In conclusion, this study demonstrates that integrating AR with AI offers a novel approach for localizing ioECoG grids during epilepsy surgery. It highlights the feasibility of using super-resolution techniques on low-resolution AR HMD cameras to accurately detect and localize ioECoG grids. While our findings are promising as it reached clinically acceptable accuracy, further research is necessary to validate these results with deformations and in clinical settings.

## Data Availability

The source code, synthetic data, and trained models used to reproduce the evaluation results in this paper are available at: GitHub Repository.
